# Association of Metabolic Markers with self-reported osteoarthritis among middle-aged BMI-defined non-obese individuals: a cross-sectional study

**DOI:** 10.1186/s40608-018-0201-9

**Published:** 2018-09-03

**Authors:** Kelsey H. Collins, Behnam Sharif, Raylene A. Reimer, Claudia Sanmartin, Walter Herzog, Rick Chin, Deborah A. Marshall

**Affiliations:** 1 Human Performance Laboratory, University of Calgary, Calgary, AB Canada; 2 Department of Community Health Sciences, University of Calgary, 3280 Hospital Drive NW, Calgary, AB T2N 4Z6 Canada; 3 Faculty of Kinesiology and Department of Biochemistry and Molecular Biology, University of Calgary, Calgary, AB Canada; 4 Health Analysis Division, Statistics Canada, Ottawa, ON Canada; 5 Department of Medicine, University of Calgary, Calgary, AB Canada

**Keywords:** Osteoarthritis, Obesity, Adipose tissue, Metabolic markers, Body fat, Population-based survey

## Abstract

**Background:**

Osteoarthritis (OA) is a chronic degenerative joint disease. While it is well-established that obesity affects OA through increased axial loading on the joint cartilage, the indirect effect of obesity through metabolic processes among the body mass index (BMI)-defined non-obese population, i.e., BMI < 30 kg/m^2^, is less known. Our goal was to evaluate the association of metabolic markers including body fat percentage (BF%), waist circumference, maximum weight gain during adulthood and serum creatinine with self-reported OA to establish if such measures offer additional information over BMI among the non-obese population between 40 and 65 years of age.

**Methods:**

Cross-sectional data from two cycles of the Canadian Health Measures Survey (CHMS) in 2007–2009 and 2009–2011 were analyzed. Sex-specific logistic regression models were developed to evaluate the association of self-reported OA with metabolic markers. Models were separately adjusted for age, BMI categories and serum creatinine, and a stratified analysis across BM categories was performed. In a secondary analysis, we evaluated the association of self-reported OA, cardiovascular diseases and hypertension across BF% categories.

**Results:**

Of 2462 individuals, 217 (8.8%) self-reported OA. After adjusting for age and BMI, those within BF%-defined overweight/obese category had 2.67 (95% CI: 1.32–3.51) and 2.11(95% CI: 1.38–3.21) times higher odds of reporting self-reported OA compared to those within BF%-defined athletic/acceptable category for females and males, respectively. BF% was also significantly associated with self-reported OA after adjusting for age and serum creatinine only among females (OR: 1.47, 95%CI: 1.12–1.84). Furthermore, among the BMI-defined overweight group, the age-adjusted odds of self-reported OA was significantly higher for overweight/obese BF% compared to athletic/acceptable BF% in both females and males. In a secondary analysis, we showed that the association of self-reported OA and hypertension/cardiovascular diseases is significantly higher among BF% overweight/obese (OR: 1.37, 95%CI: 1.19–3.09) compared to BF% athletic/acceptable (OR: 1.13, 95%CI: 0.87–2.82).

**Conclusion:**

Our results provide corroborating evidence for a relationship between body fat and OA in a population-based study, while no significant independent correlates were found between other metabolic markers and OA prevalence. Future investigation on the longitudinal relationship between BF and OA among this sub-population may inform targeted prevention opportunities.

**Electronic supplementary material:**

The online version of this article (10.1186/s40608-018-0201-9) contains supplementary material, which is available to authorized users.

## Introduction

Osteoarthritis (OA) is a multifactorial degenerative joint disease affecting more than 1 in 8 individuals worldwide [1]. OA is among the fastest growing causes of loss in Disability Adjusted Life Years (DALYs) [2] mainly because of the aging population and increasing incidence of obesity [1, 2]. Obesity is conventionally measured using body mass index (BMI), and several studies have shown that individuals with obesity according to a BMI ≥ 30 are at higher risk for developing radiographic and symptomatic OA [1, 3]. Previous studies have shown that measurements of body composition and fat distribution may offer no advantage over BMI in assessment of risk for severe radiographic knee OA among the obese population [4, 5]. However, there is a paucity of research evaluating the independent association of metabolic markers and OA prevalence among a non-obese population [6].

Previous studies suggested that the initiation and progression of OA results from a complex interaction between mechanical axial loading on joints and various lipid, metabolic, and humoral risk factors [7–10]. Furthermore, recent findings on the significant association of body fat distribution with OA in non-weight bearing joints suggests that obesity-associated systemic factors could play an important role in the development and progression of OA [11–15]. Brasnjevic et al. [16] showed that abdominal obesity was associated with radiographic progression of knee OA, and in a large Japanese cohort study, accumulation of metabolic syndrome components was related to the incidence and progression of knee OA components [17]. Preclinical models in rats also suggested a role of chronic inflammation, likely from metabolic stress, in OA onset and progression [18–22].

Muscle weakness has also been identified as a key factor affecting OA onset and progression that further emphasizes the role for body composition, body fat distribution and OA development [23–27]. In a population-based study, Ding et al. [24] noted a link between muscle weakness and body fat with OA, and found that the additive effects of muscle weakness and body fat increase the relative risk of OA compared to age and sex matched controls. Serum creatinine levels have been used as a surrogate measure for muscle mass in patients with chronic diseases [28, 29] and are also associated with lean mass in healthy individuals [30].

Several studies have also demonstrated that overweight (BMI between 25 to 30 kg/m^2^) during middle adulthood may play an important role in OA’s initiation and progression [31, 32]. Furthermore, OA management strategies and obesity prevention interventions [33] are most effective in the long-run when they are targeted to patients with overweight (BMI-defined non-obese) at early stages of the disease process [34]. As such, Manninen et al. [35] highlights the need to identify candidates for OA prevention and management strategies in the middle-aged and the non-obese.

The purpose of this study was to investigate the independent association between metabolic markers and self-reported OA among middle-aged BMI-defined non-obese individuals (40 to 65 years of age and BMI < 30 kg/m^2^). As such, we focus on measurements that are low-cost, ready to be implemented, and widely used in large population-based study cohorts including skinfold measured body fat, waist circumference and weight gained since adulthood. As the study population may not be assessed for OA prevalence at this time, the use of such metrics may be a feasible approach to support data collection and potential screening for adiposity and OA prevalence in this population who may not otherwise be assessed [35].

## Methods

### Data source and population

The Canadian Health Measures Survey (CHMS) is a bi-annual survey of Canadians health and health habits [36]. In the CHMS, Statistics Canada collects data from a nationally representative sample of the Canadian population aged 6–79 years living in private households in which approximately 96% of Canadians were represented [37]. CHMS is the first comprehensive and representative direct health measures study in Canada since the 1978–1979 Canada Health Survey [37].

The survey involved two components: an interview in the respondent’s home and a visit to a mobile examination center for a series of physical and clinical measurements. In all cycles of CHMS, data were collected at 15 sites across Canada. The interview included questions from respondents about a range of chronic conditions, defined as a condition diagnosed by a health professional and lasting, or expected to last, more than 6 months including OA, type 2 diabetes, and heart disease. Clinical and physical measures at an examination center included anthropometry, blood pressure, oral health examination, and blood and urine specimens [37]. Ethics approval to conduct the CHMS was obtained from Health Canada’s Research Ethics Board. Informed written consent was obtained from all adult respondents.

### Sample selection

**Fig. 1:**
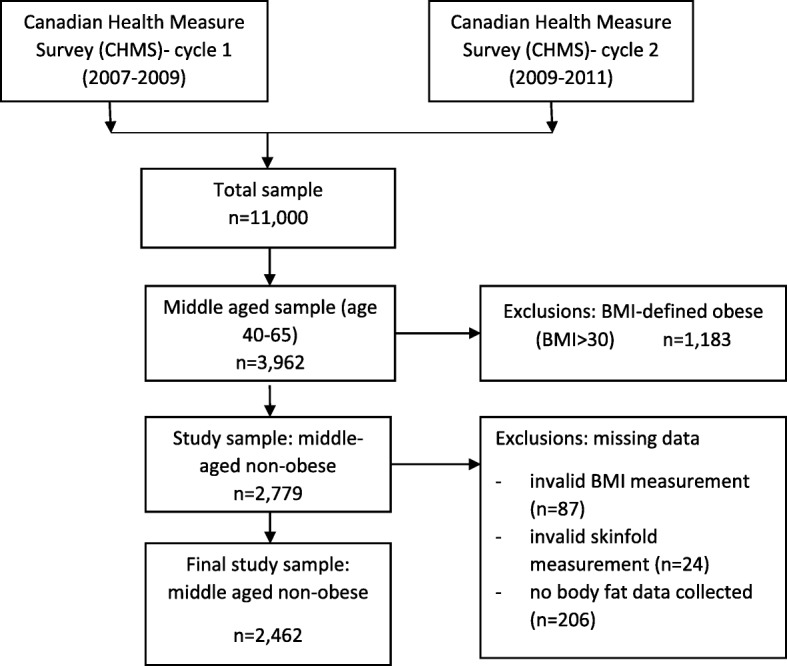
Flow chart of the study sample selection

In this study, we used two cycles of the CHMS: 2007–2009, and 2009–2011. Health measures were included from both cycles. As our aim was to evaluate the relationship between body composition measurements and OA in non-obese middle-aged individuals, we included individuals between ages 40 to 65 years with BMI < 30 kg/m^2^. Individuals were not included if data were missing due to invalid BMI measurement, invalid skinfold measurements or if there were no body fat data collected. Figure 1 depicts the diagram of the sample selection from two cycles of CHMS included in this study.

### Primary outcome

Osteoarthritis was determined from a patient reported response to the survey question about the physician-diagnosed arthritis: what kind of arthritis do you have? 1. Rheumatoid arthritis, 2. Osteoarthritis, 3. Rheumatism, or 4. Other. As a result, self-reported physician diagnosed OA was used as the outcome.

### Metabolic markers

The measures used in this study were selected according to the metrics used in the literature for OA patients [4–7], which are low-cost, readily available and have been validated in population-based studies. Height was measured to the nearest 0.1 cm and weight was measured to the nearest 0.1 kg by standard devices on each mobile examination center [36]. BMI was calculated and classified according to standardized thresholds: normal (18.5 to 24.9 kg/m^2^), overweight (25.0 to 29.9 kg/m^2^) [36]**.** To determine the body fat, body density was first calculated using the summation of skinfold measures at four sites (triceps, biceps, subscapula, and iliac crest). Once body density was determined, body fat was calculated using a general sex-specific equation previously derived and validated by Durnin et al. [38]. Individual body fat percentages (BF%) were grouped into two body fat categories according to their distributions: athletic/acceptable (males: ≤20% females ≤30%), or overweight/obese (male > 20% females > 30%). This skinfold caliper technique is only validated for use in a population with BMI < 30 kg/m^2^, and as such, evaluating it excludes individuals who are obese by BMI [7].

Waist circumference was measured between the last rib and the top of the iliac crest after an expiration to the nearest 0.1 cm [36]. Waist circumference categories was defined according to standard thresholds used for females (> 88 cm and < 88 cm) and those for males (> 102 cm and < 102 cm) [39]. Maximum weight gain during adulthood was derived by subtracting “most adult weight ever” from the “weight at 18 years of age”. Previous studies have also examined maximum weight gain during adulthood in assessing risk of chronic diseases [40]. Laboratory Serum creatinine measures (μmol/L) were used as a surrogate for muscle mass [41]. Threshold for definition of high and low value of serum creatinine was used from [41] in which high values were defined as those with ≥65 μmol/L among males and ≥ 73 μmol/L among females.

### Comorbidity assessments

We also extracted data on cardiovascular (CVD)-related comorbidities which included self-reported measures on history of heart disease, heart attack or high blood pressure. These were assessed using a response to three CHMS household questionnaire: (i) CCC_Q61: Do you have heart disease (1) Yes (0) No or (ii)CCC_Q63: Have you had a heart attack? (1) Yes (0) No, or (iii) CCC_Q64: Do you have high blood pressure?, (1) Yes (0) No. Clinical measurement for blood creatinine was also included as an indirect assessment of muscle mass (μmol/L). The sample size for responses to other chronic conditions including diabetes was small, and violated the minimal cell count prescribed by Statistics Canada, and therefore they were not included in the analysis.

### Statistical analysis

All variables were assessed to ensure similarities in questions and responses. Cycles 1 and 2 were combined using methods described in the Statistics Canada documentation for combining the two CHMS datasets [36]. Quality assurance and quality control measures were previously performed independently on both CHMS datasets to minimize systematic bias [37]. Categorical variables were examined using frequency tables, and continuous variables were evaluated using summary statistics. If laboratory values were missing for blood creatinine, they were imputed using the level of detection limit divided by 2 [36]. Univariate analyses were conducted to investigate crude associations between the covariates and self-reported OA. In univariate analysis, *p*-values were based on an adjusted Pearson chi-squared test for independence (categorical variables), or a Bonferroni-adjusted Wald F-test (continuous variables). All results are weighted and standard errors are estimated using bootstrapping to account for the survey design effects of the CHMS. Kolmogorov–Smirnov tests were also performed to assess the normality of data for metabolic markers (*p* < 0.05). Multicollinearity diagnostics were performed by calculating variance inflation factor (VIF), which quantifies the severity of multicollinearity in an ordinary least squares regression analysis. VIF of BMI in all models were less than 2.6, which is below the threshold of 4 to represent multicollinearity.

Sex-stratified logistic regression models were developed to evaluate the association of self-reported OA with the five metabolic markers in this study. Models were separately adjusted for age, age and BMI, and age and blood creatinine levels in order to detect additional correlations that may exist between metabolic markers and self-reported OA. We further performed a stratified analysis across BMI groups to evaluate the independent association of BF% and self-reported OA among the BMI-defined overweight group and assess the additional information BF% could potentially provide other than those gained from BMI. In a secondary analysis, we developed logistic regression models to assess the association between OA and hypertension/CVD across BF% categories to identify the possible mediating role of the body fat on the association of OA and hypertension/CVD. Due to the small sample size within each cell for logistic regression modelling, and the limited age range in this sample of interest, no interactions with age or sex were included. All results were weighted according to the survey weights to represent the population-based sampling scheme of the CHMS. To account for survey design effects of the CHMS, standard errors, coefficients of variation, and 95% confidence intervals were estimated using the bootstrap technique (500 replications) provided in the CHMS documentation[36].

## Results

A total of 2462 individuals between 40 to 65 years of age with BMI < 30 were selected for the final analysis, of which 217 (8.8%) reported OA (Fig. 1). Kolmogorov–Smirnov tests revealed that BMI, BF%, waist circumference and maximum weight gain data were normally distributed (*p* > 0.05). As shown in Table 1, the proportion of females was significantly higher among the individuals with self-reported OA (77%, SE: 3.5) compared to the non-OA (49.5%, SE: 0.94). The average BF% of individuals with self-reported OA was also higher than those of the non-OA group among both men [28.1%, SE:0.5) vs. (23.2%, SE:0.3), *p* < 0.01] and women [30.2%, SE: 0.3) vs. 33.9%, SE:0.2, p < 0.01], while waist circumference was significantly higher only among men [89.0 cm (SE:0.5) vs. 93.0 cm (SE:1.1), p < 0.01]. Furthermore, higher proportion of the self-reported OA indicated at least one CVD-related comorbidity compared to that of the non-OA sample among males [12.4%, SE: 2. vs. 4.9%, SE: 0.6, p < 0.01] and females [9.1% (SE: 2.1) vs. 3.8% (SE:0.5)] and had lower blood creatinine among males [78.1 μmol/L (SE: 1.0) vs. 82.4 μmol/L (SE: 0.5), p = < 0.01] and females [67.3 μmol/L (SE:1.0) vs. 72.8 μmol/L (SE:0.5), *p* = < 0.01]. The detailed results for the count of individuals within each BF%, BMI and sex groups are provided in Additional file 1: Table S1.

**Table 1:** Characteristics of individuals with self-reported osteoarthritis (OA) compared to individuals without self-reported OA^a^

Characteristics ^b^	Overall sample	No Self-Reported OA	Self-Reported OA	*p*-value^d^
	*n* = 2462	*n* = 2245	*n* = 217	
Demographics				
Age (years), mean (SE^c^)	52.6 (0.3)	52.2 (0.2)	57.3 (0.6)	< 0.001
Females, % (SE)	51 (1.2)	49.0 (0.9)	77.0 (3.5)	< 0.001
Metabolic Markers				
*Females (n = 1267)*				
Body Fat Percentage, mean (SE)	30.58 (0.3)	30.20 (0.2)	33.90 (0.3)	< 0.01
Body Mass Index (kg/m^2^), mean (SE)	26.33 (0.5)	26.20(0.4)	27.25(1.0)	0.112
Waist Circumference (cm), mean (SE)	78.49(0.4)	78.23(0.4)	80.31(0.9)	0.08
Maximum Weight Change (kg), mean (SE)	19.06(0.4)	18.90(0.5)	20.70 (0.9)	0.12
Serum Creatinine (μmol/L), mean (SE)	71.6 (0.6)	72.8 (0.5)	67.3 (1.0)	< 0.01
Hypertension/CVD^e^, % (SE)	3.9 (0.6)	3.8 (0.5)	9.1 (2.1)	< 0.01
*Males (n = 1195)*				
Body Fat Percentage, mean (SE)	23.67 (0.4)	23.2(0.3)	28.12(0.5)	< 0.01
Body Mass Index (kg/m^2^), mean (SE)	27.47 (0.6)	27.10 (0.5)	28.90 (1.2)	0.318
Waist Circumference (cm), mean (SE)	89.53 (0.6)	89.00 (0.5)	93.00 (1.0)	< 0.05
Maximum Weight Change (kg), mean (SE)	16.46 (0.6)	16.30 (0.5)	18.10 (1.1)	0.231
Serum Creatinine (μmol/L), mean (SE)	80.3 (0.5)	82.4 (0.5)	78.1 (1.0)	< 0.01
Hypertension/CVD^e^, % (SE)	5.1 (0.7)	4.9 (0.6)	12.4 (2.0)	< 0.01

^a^ All results are weighted according to the sample weights of the Canadian Health Measure Survey (CHMS); ^b^ Anthropometric, laboratory values, and percentage with comorbidity for patients with and without self-reported OA. ^c^ Standard error estimates were generated using 500 bootstrap replications. All statistics were evaluated at α = 0.05. ^d^ *P*-values are used for comparing measures across self-reported OA and non-OA; for categorical variables, *p*-values were based on an adjusted Pearson chi-squared test for independence, and *p*-values for continuous variables were based on an adjusted Wald F test. ^e^ Maximum Weight Change as an adult from 18-years-old; ^f^Hypertension/CVD: Self-reported hypertension or Cardiovascular Disease

As shown in Table 2 for the result of the multivariate analysis, higher BF% and waist circumference categories were significantly associated with self-reported OA, independent of age, for both males and females (*p* < 0.05). Those within the BF%-defined obese/overweight category had higher odds of self-reported OA compared to those within the BF%-defined athletic/acceptable category for females (OR: 3.2, 95% CI: 1.25–4.4) and males (OR: 1.82, 95% CI: 1.15–3.58) after adjusting for age. Similarly, those in the high waist circumference category had significantly higher odds of self-reported OA compared to those in the low category for both females (OR: 2.62, 95% CI: 1.51–3.01) and males (OR: 2.21, 95% CI:1.3–3.81) after adjusting for age. Furthermore, among males, each kilogram of maximum weight gained was associated with 3% higher odds of self-reported OA independent of the current age (OR: 1.04, 95% CI: 1.02–1.76).

**Table 2:** Odds ratios (and 95% confidence intervals) from logistic regressions for associations of metabolic markers with self-reported OA^a^

	Models	Categories	Adjusted models (by age)	Adjusted models (by age, BMI)	Adjusted models (by age, serum creatinine)
Female (*n = 1267*)	Model 1. BMI categories	Underweight/normal (< 25 kg/m^2^)	Ref	–	Ref
		Overweight (25–30 kg/m^2^)	1.53 (0.65, 2.13)	–	1.32 (0.87,2.21)
	Model 2. BF%^b^ categories	Athletic/ Acceptable	Ref	Ref	Ref
		Overweight/obese	3.12 (1.25,4.4)*	2.67 (1.32,3.51)*	1.47 (1.12,1.84)*
	Model 3. Waist circumference (cm)	< 88 cm	Ref	Ref	Ref
		> 88 cm	2.62 (1.51,3.01)*	0.91 (0.80,2.64)	1.27 (0.91,2.14)
	Model 4. Maximum Weight change (kg)^c^	–	1.03 (0.91,1.43)	1.07 (0.96,2.46)	1.03 (0.82,2.68)
	Model 5. Serum Creatinine	≥65 μmol/L	Ref	Ref	–
		< 65 μmol/L	1.21 (0.87,2.45)	0.87 (0.45,1.97)	–
Male *(n = 1195*)	Model 1. BMI categories	Underweight/normal (< 25 kg/m^2^)	Ref	–	Ref
		Overweight (25–30 kg/m^2^)	0.92 (0.75,1.91)	–	1.43 (0.93,2.45)
	Model 2. BF%^b^ categories	Athletic/ acceptable	Ref	Ref	Ref
		Overweight/obese	1.82 (1.15,3.58)*	2.11 (1.38,3.21)*	1.12 (0.78,1.93)
	Model 3. Waist circumference (cm)	< 102 cm	Ref	Ref	Ref
		> 102 cm	2.21 (1.3,3.81)*	0.91(0.83,1.54)	1.08 (0.91,1.72)
	Model 4. Maximum Weight change ^c^(kg)	–	1.04 (1.02,1.76)*	1.05 (0.87,2.32)	0.29 (0.12,2.16)
	Model 5. Serum Creatinine	≥73 μmol/L	Ref	Ref	–
		< 73 μmol/L	1.97 (0.91,2.89)	0.91 (0.35,2.1)	–

^a^ All results are weighted according to the sample weights of the Canadian Health Measure Survey (CHMS); ^b^ Body Fat percentage (BF%);^c^ Individual body fat percentages were grouped into three body fat categories: athletic/good (males: < 14% females < 23%), acceptable (males 15–20%, females 24–30%), or overweight/obese (male > 21% females > 31%).; ^d^ Maximum Weight change as an adult from 18-years-old (kg); * p-value< 0.05

As shown in Table 2, only the BF%-defined obese/overweight category was associated with self-reported OA independent of BMI and age. The adjusted odds of self-reported OA was higher among overweight/obese BF% compared to athletic/acceptable category among females (OR: 2.67, 95%CI:1.32–3.51) and males (OR:2.11, 95%CI:1.38–3.21) after adjusting for age and BMI. Furthermore, BF% was significantly associated with self-reported OA after adjusting for age and serum creatinine only among females (OR: 1.47, 95%CI: 1.12–1.84).

BMI-stratified analysis in Table 3 revealed that among the BMI-defined overweight group, the age-adjusted odds of self-reported OA was significantly higher for overweight/obese BF% compared to athletic/acceptable BF% in both females (OR:1.84, 95% CI:1.18–3.19) and males (OR:1.32,95% CI:1.12–2.79). The association between OA and BF% categories was not significant among the BMI-defined underweight/normal group.

**Table 3:** Age-adjusted odds ratio (and 95% confidence intervals) for self-reported OA across BF% categories stratified by BMI groups and sex

	BMI	Underweight/Normal	Overweight
	Body Fat Percentage	(BMI < 25 kg/m^2^)	(BMI 25–29.99 kg/m^2^)
Female	Athletic/Acceptable	Reference	Reference
	Overweight/Obese	1.92 (0.81,2.87)	1.84 (1.18,3.19)*
		*(n = 711)*	*(n = 556)*
Male	Athletic/Acceptable	Reference	Reference
	Overweight/Obese	1.41 (0.61,2.39)	1.32 (1.12,2.79)*
		*(n = 394)*	*(n = 801)*

All four models were adjusted for age; Individual body fat percentages were grouped into three body fat categories: athletic/good (males: < 14% females < 23%), acceptable (males 15–20%, females 24–30%), or overweight/obese (male > 21% females > 31%).;* p-value< 0.05

According to our secondary analysis for association of OA and CVD across BF% categories, the age and sex–adjusted odds of self-reported OA was 37% (OR:1.37, 95%CI: 1.19–3.09) higher in those with CVD compared to those without CVD among the overweight/obese BF% category, while the OA and CVD association was not significant among those with athletic/acceptable BF% (OR: 1.13, 95% CI:0.87–2.82).

## Discussion

In this study, BF% measured by skinfold calipers provides important information with respect to self-reported OA among a Canadian sample of middle-aged BMI-defined non-obese individuals (40 to 65 years of age and BMI < 30 kg/m^2^) that is not necessarily captured by BMI alone. According to these results, BF% categories are independently associated with self-reported OA while adjusting for BMI and age, and there is a significant difference between the odds of OA across body fat categories for those within the BMI-defined overweight category (BMI between 25 and 30 kg/m^2^) in both male and female models. No independent correlates were found between other metabolic markers used in this study and self-reported OA among the study population.

Our results, however, demonstrate a univariate relationship between decreased serum creatinine, an indirect measurement of muscle mass [29] with increased OA. Furthermore, in the female-specific model, the odds of self-reported OA was significantly higher among obese/overweight BF% compared to athletic/acceptable BF% when adjusting for age and blood creatinine. This suggests that body fat may provide independent information other than that gained from serum creatinine regarding the relations between body composition and OA. Our findings suggest that both increased body fat and decreased lean mass may be characteristic of OA, but the causal contribution of these metabolic abnormalities to OA have yet to be directly tested and explored [29]. In a previous study, the combined effects of reduced lean mass with increased fat mass were shown to be associated with elevated risk of OA compared to obesity alone [42]. Results from the present study provide cross-sectional evidence for an association between obesity and OA among the middle-aged non-obese population. Longitudinal studies are needed to confirm these associations.

We further showed that the age and sex-adjusted association of self-reported OA and CVD was significantly higher in the obese/overweight BF% group compared to the association observed in athletic/acceptable BF% group. This highlights the importance of BF% in identifying those with multi-morbidity among OA population. Similar results were reported in a recent large Japanese cohort study [17] where accumulation of metabolic syndrome components were shown to be related to the incidence and progression of knee OA components. OA individuals with overweight or obesity have shown to demonstrate higher abdominal adiposity (waist circumference) [25], increased waist-to-height ratio, increased rates of high blood pressure, high cholesterol, and higher body fat compared to OA patients with athletic/acceptable BF% [43]. A recent study evaluated the cross-sectional association between metabolic markers and OA among the BMI-defined non-obese and showed that skinfold BF% is associated with higher OA prevalence [44] which corresponds to our results. Brasnjevic et al. [16] showed that abdominal obesity was associated with radiographic progression of knee OA.

While several studies showed that body fat distribution does not provide additional information other than those gained through BMI among individuals with severe knee OA [4, 5], there is a scarcity of research that individually assesses the non-obese population in this context. Our study demonstrates that measuring body fat may provide additional information that is supplementary to, or independent of, BMI with regards to OA prevalence among the middle-aged non-obese OA patients. Understanding the association of BF% and other metabolic markers with OA within a non-obese population may help improve the development of screening policies for prevention and management strategies in OA [33]. For instance, the discordance between BMI and body fat is predominantly observed in women with “healthy” BMIs (BMI ≤24.99) but who are overweight or obese based on metabolic markers such as levels of body fat (BF% ≥ 31%) [21, 23, 24] or abdominal obesity measured by waist circumference [8, 9]. Given that these metabolic markers have shown to be significantly associated with OA progression [16] and health outcomes [17], such women would traditionally be medically managed as healthy, and as such, and may miss OA prevention opportunities even though they may be at increased risk.

As our data are representative of the Canadian population, they are likely generalizable to other countries with population characteristics similar to that of Canada. There are known differences in body fat and body composition across races and ethnicities [45], and the predictability of body fat by the equations used by the CHMS dataset. The data presented in this study reflects the ethnic diversity of Canadians. OA affects more than 4.6 million Canadians [46], and it is estimated that 1 in 4 Canadians will have OA by 2040 due to aging, increased longevity of the population and the obesity epidemic [46]. Countries with a homogeneous racial or ethnic population should validate these findings in their given population, as differences in fat storage between races have been previously reported [47].

A body fat classification system from Durnin et al. [38] was used in this study that divided individuals into categories based on BF% and sex. Other body fat categorization schemes have been developed, but include age [48]. Specifically, the World Health Organization criteria incorporates increased BF% in the “acceptable” category for each increasing age group, which makes isolating the individual effect of body fat on OA challenging [49]. As the goal of this study was to evaluate the effects of discrete BF% values on OA, a fixed sex-specific classification of BF% was used. Body fat changes with age [50], and both age and sex are strongly associated with OA [1]. To compensate for this, our models were adjusted for age and stratified by sex.

There are several limitations to this work. One significant limitation of the CMHS dataset is that it does not include data on previous injury or family history of OA. However, as previously mentioned, there are several accepted primary risk factors calling for sub classification, or subtypes of OA to define a “profile” that describes each of these subtypes [25]. Therefore, we speculate that the trend toward an increased odds ratio of athletic/acceptable BF% individuals for OA demonstrated here may be related to these factors that were not measured. As all individuals included in this study had a BMI < 30 kg/m^2^, it is possible that they demonstrate a “pre-metabolic syndrome” phenotype but may still have metabolic abnormalities contributing to their OA. Here, we were unable to evaluate all metabolic parameters of interest in the logistic regression models because of weighting limitations associated with combining fasted lab values from the CHMS Statistics Canada dataset. Due to the limited sample size of our study, we also did not adjust for a variety of risk factors that are known to be related to OA (socioeconomic status, ethnicity, smoking), but that would be useful to consider in future investigations. Additionally, the limited age group evaluated did not allow for us to evaluate these relations across the weight or age spectrum. There are also limitations associated with the use of skinfold anthropometry; it may underestimate BF% when compared to other methods such as dual energy x-ray absorptiometry as the former does not account for intra-abdominal or visceral adipose tissue stores [51]. Consequently, skinfold anthropometry lacks appropriate population-specific cut-off values to identify health risks among individuals with obesity [51]. Lastly, a substantial limitation of this work was the binary and self-reported nature of the primary outcome measure, OA. This analysis is based on an outcome variable acquired from self-report data. However, we suggest that the preliminary context of this study and information gained from this analysis justifies the use of this outcome.

Future work should longitudinally evaluate the association of body fat, lean mass, muscle strength, pain and structural graded MRI Osteoarthritis Knee Score [4] or radiographic Kellgren-Lawrence outcomes [4] to better understand implications of metabolic risk factors, adiposity, and osteoarthritis severity among the non-obese population. Clinically, better measures of body fat or body composition (e.g., DXA [47]) could be used to understand risk of developing OA according to body fat measurements in patients across the spectrum of body composition. The presence of an OA subtype may be supported by a relationship between increased body fat, indirect measures of body composition, elevated CVD risk, and OA.

## Conclusion

This study demonstrates that measuring body fat may provide additional information over and above BMI with regards to OA prevalence among the middle-aged non-obese population. However, no independent correlate was found between other metabolic markers used in this study and the self-reported OA among the study population. Given the rise in prevalence of OA among the middle-aged population [46] and the increased effectiveness of prevention interventions among the BMI-defined non-obese population [34], this study highlights the need to better understand the interrelation of body fat and risk of OA among the BMI-defined non-obese population. Our results pave the way to further explore the use of low-cost, readily available, population-based metrics for metabolic markers as a primary care screening tool among younger non-obese individuals, who may not otherwise be assessed for OA risk or musculoskeletal compromise.

## Additional file

Additional file 1: BMI and body fat percentage classification. The supplementary data provides a summary of the sample data for BMI and body fat percentage classification across males and females. (DOCX 14 kb)

## Abbreviations

BF: Body Fat
BMI: Body Mass Index
CHMS: Canadian Health Measures Survey
CVD: Cardiovascular diseases
OA: Osteoarthritis
SE: Standard Error
