# Supplementary material for: Association of Metabolic Markers with self-reported osteoarthritis among middle-aged BMI-defined non-obese individuals: a cross-sectional study
Source: BMC Obes. 2018 Sep 3;5:23. doi: 10.1186/s40608-018-0201-9 (PMC6120068; doi:10.1186/s40608-018-0201-9)
Supplement: Supplementary file 1 — BMI and body fat percentage classification. The supplementary data provides a summary of the sample data for BMI and body fat percentage classification across males and females. (DOCX 14 kb) [file 40608_2018_201_MOESM1_ESM.docx]

**Supplementary Material**

**Table A1.** BMI and body fat percentage classification are discordant for approximately 30% of the total middle aged, non-obese population (BMI<30 Kg/m^2^ and age between 40 to 65 years)

| **Frequency**  **Body Fat Percentage Categories**  **(n=2,462)** | **BMI Categories** | | | | | | | | | |  |
| --- | --- | --- | --- | --- | --- | --- | --- | --- | --- | --- | --- |
|  | **Underweight/Normal**  **(BMI < 25 kg/m^2^)** | | | | | **Overweight**  **(BMI 25 – 29.99 kg/m^2^)** | | | | | **P-value** |
| **Female (n = 1267)** | ***n*** | % | CI Lower Limit | CI Upper Limit | (CV %) | ***n*** | % | CI Lower Limit | CI Upper Limit | (CV %) | p<0.001 |
| Athletic/Acceptable | ***285*** | 21 | 240 | 335 | 8.1 | ***21*** | 2 | 13 | 34 | 24 |  |
| Overweight/Obese | ***416*** | 30 | 358 | 478 | 7.0 | ***581*** | 43 | 528 | 636 | 4.5 |  |
| **Male (n = 1195)** | ***n*** | % | CI Lower Limit | CI Upper Limit | (CV %) | ***n*** | % | CI Lower Limit | CI Upper Limit | (CV %) | p<0.001 |
| Athletic/ Acceptable | ***177*** | 13 | 145 | 214 | 9.5 | ***179*** | 14 | 146 | 218 | 9.7 |  |
| Overweight/Obese | ***151*** | 12 | 110 | 205 | 15.0 | ***672*** | 52 | 609 | 734 | 4.5 |  |
| **Overall (n = 2462)** | ***n*** | % | CI Lower Limit | CI Upper Limit | (CV %) | ***n*** | % | CI Lower Limit | CI Upper Limit | (CV %) | p<0.001 |
| Athletic/Acceptable | ***462*** | 17 | 397 | 534 | 7.2 | ***200*** | 8 | 163 | 243 | 9.7 |  |
| Overweight/Obese | ***567*** | 21 | 487 | 655 | 7.2 | ***1253*** | 47 | 1165 | 1341 | 3.4 |  |

Body fat percentage categories versus body mass index (BMI) classification by sex. % show the number out of overall sex-specific sample. Approximately 26% of men and 31% of women demonstrate discordance between BMI and percent body fat classification. P-value based on an adjusted Pearson chi-squared test for independence. Red Values indicate estimates that do not meet Statistics Canada’s quality standards of coefficient of variation >33.3%.
